# Prospects and challenges of US and CEUS

**DOI:** 10.1186/1470-7330-14-S1-O13

**Published:** 2014-10-09

**Authors:** Olivier Lucidarme, Jean Michel Correas

**Affiliations:** 1 Service d'imagerie polyvalente, Groupe hospitalier Pitié–Salpêtrière, AP-HP, France; 2 Service de radiologie adulte, Hôpital Necker, AP-HP, France

## 

Ultrasound is already a major technique to study focal liver lesions. Particularly since the arrival of the ultrasound contrast agents which allows a sensitivity greater than 90% for characterizing incidentally detected focal liver lesions in adults in whom an unenhanced ultrasound scan is inconclusive or with inconclusive MRI/CT. However the role of ultrasound will probably rise even more in the near future because of rapid software and hardware developments.

Future prospects are:

1) The development of image fusion and navigation technology combining US and CT or MRI to improve the possibility to perform difficult percutaneous ultrasound guided biopsy or thermo ablation procedures with a higher rate of success.

2) The development of 3D imaging technology in ultrasound to improve liver tumour response assessment particularly by means of a combination with ultrasound contrast agents. The challenge will be to be able to reach a real time 3D imaging through matrix technologies also capable of handling contrast agents enhanced modes in order to get enhancement curves of the whole tumour with a adapted temporal resolution.

3) The development of targeted imaging through targeted microbubbles against a variety of targets located on the vessel wall. Indeed contrast enhanced ultrasound is probably the second more sensitive technique to the presence of a small amount of targeted contrast compound after PET. Many feasibility studies on animal models have already been conducted and currently a hypo allergenic targeted microbubble that includes in its membrane a heterodimer peptide having a high affinity to VEGFR2 (BR55) is being tested in humans.

Beside CT and MRI, US must be also considered as a major technique that has much to offer particularly in liver imaging and the near future of ultrasound is undoubtedly exciting.

